# ‘Show Us a Kiss!’: The Everyday Sexual Harassment Experiences of Female Lesbian, Bisexual, and Queer Students

**DOI:** 10.1177/10778012231166399

**Published:** 2023-04-05

**Authors:** Gemma L. Witcomb, Charlotte Cooper

**Affiliations:** 1 School of Sport, Exercise and Health Sciences, 5156 Loughborough University, Loughborough, LE11 3TU UK

**Keywords:** LGBT+, harassment, heterosexism, university, education

## Abstract

This study explored the experiences of lesbian, bisexual, and queer (LBQ) students’ (*N* = 9, aged 19–24) of sexual harassment via semistructured interviews. Data were analysed using a thematic analysis. Three overarching themes emerged: (a) the paradox of men's unwanted sexual attention, (b) the negative impact on relationships, and (c) the LGBTQ* community as a refuge. The women reported enduring unwanted heteronormative sexual attention, and heterosexist and homophobic harassment which forced some to conceal their sexuality. Support for the LGBTQ* community was central in developing confidence to call-out harassment. The findings call for the inclusion of LBQ-specific messages in sexual violence awareness and prevention interventions.

## Introduction

Sexual harassment is an everyday phenomenon in the lives of young women which is normalized from an early age (Berman et al., 2000). Recently, it has become increasingly recognized that sexual harassment is a widespread problem, particularly within the university student population (Towl & Walker, 2019). However, this recognition is often presented through a largely heterosexist lens, of heterosexual women being harassed by heterosexual men (Lazard, 2020). This heteronormative view fails to acknowledge the differing experiences of lesbian, bisexual, and queer (LBQ) women, not only in the nature of, and reaction to, harassment from heterosexual men, but also in instances of harassment by other LBQ women (Cipriano et al., 2022), and by doing so, it erases the experiences of LBQ women from the mainstream narrative.

Gelfand et al. (1995) describe three types of sexual harassment: gender harassment, unwanted sexual attention, and sexual coercion. Gender harassment refers to behaviors that communicate derogatory attitudes about women such as sexist jokes, gestures, or images. Unwanted sexual attention includes verbal and nonverbal behaviors like catcalling, groping, and persistent nonreciprocal requests for dates. Finally, sexual coercion represents threats or bribes conditional to sexual cooperation. Popular sociocultural and feminist theory situates sexual harassment as a gender-based phenomenon (Fileborn, 2014; MacKinnon, 1979) considered to be a (heteronormative) mechanism of power motivated by men's desire to reinforce their superior sex-based status (Berdahl, 2007). This is borne out in the prevalence data which situate women as more likely to be a victim than men. For example, a 2020 UK Government Equalities Office survey of 12,131 individuals found that 34% of men had experienced an incident of sexual harassment within the last 12 months compared to 51% of women (Adams et al., 2020). Similarly, a 2017 survey of 6,026 individuals in the UK found that 80% of men responded ‘no’ to having ever experienced harassment, but only 58% of women responded in the negative (ComRes, 2017). In addition, gay men are more likely to experience sexual harassment than heterosexual men (Chen et al., 2020). However, heterosexual men may be less likely to perceive behaviors as harassment compared to women and gay men and therefore be less likely to report it (Clarke, 2022) or be less likely to report it due to heterosexist fears of being perceived as unmasculine (Schermerhorn & Vescio, 2022).

While women are disproportionately affected by harassment at all stages in life (Bondestam & Lundqvist, 2020; Latcheva, 2017), young women attending universities have been identified as a particularly high-risk group (Mellgren et al., 2018). This is because universities remain largely patriarchal environments where harassment by males is a common, daily occurrence (Diaz-Fernandez & Evans, 2020). Due to the unique context in which students are living, learning, and socializing within the same surroundings, pervasive harassment can permeate a woman's entire environment (Whitfield & Dustin, 2016).

The prevalence of university harassment is difficult to establish since empirical studies use a range of definitions. In the UK, the National Union of Students’ research puts this estimate at 68% in 2010 (Smith, 2010), rising to 75% in 2019 (NUS, 2019). In all cases, women reported being subjected to sexual comments and noises as well as physical and unwanted sexual attention (e.g., inappropriate touching and groping) both on and off campus. In the most recent 2019 report, over a third (37%) of women had experienced unwanted sexual remarks via social media or text messages. However prevalence data and outcome studies tend to focus on heterosexual samples or neglect to mention the sexual orientation of participants (Fedina et al., 2018). When sexuality is included it is clear that sexual minority students are disproportionately exposed to sexual harassment compared to their heterosexual peers (Martin-Storey et al., 2018; Murchison et al., 2017; Wood et al., 2018).

It is helpful to apply Fileborn's (2014) understanding of the intersecting nature of unwanted sexual attention and heterosexist and homophobic violence. Whilst these are often conceptualized as distinct constructs, the accounts of sexual minority women reveal that they often represent overlapping experiences (Fileborn, 2014). Homophobia and heterosexism remain rife in university culture (Ellis, 2009; NUS, 2014; Phipps & Young, 2013), placing sexual minority women in a particularly vulnerable position. Their gender identity as a woman makes them a likely target of harassment, but they may also additionally be viewed and treated as sexual objects due to their participation in same-sex relationships. Sexual minority women report having to endure aggressive flirtation from heterosexual men who ignore and undermine their sexual exclusivity to one another (Hequembourg & Brallier, 2009). This form of unwanted sexual attention is unlikely to occur if a woman is with a male partner. While many aspects of harassment are common in accounts of both heterosexual and sexual minority women (e.g., involving alcohol, occurring in public spaces, and relentless sexual requests; Menning & Holtzman, 2014), others will be uniquely related to their perceived sexuality. Thus there is a need for theoretical understandings to better consider the role of sexual identity in the lived experience of unwanted sexual attention.

Indeed, sexual minorities are a marginalized group and are vulnerable to additional stressors related to their group status that can impact on mental well-being. Recently, it was reported that young (aged 18–24) sexual minority females in England are five times more likely to suffer from chronic mental health problems compared to their heterosexual counterparts (MacCarthy et al., 2022). Meyer's (2003) minority stress theory (MST) posits that there are four interrelated minority stress processes through which sexual minority status influences well-being: prejudice events, stigma (expectations of rejection and discrimination), concealment, and internalized homophobia. Since over half of all sexual minority students report concealing their sexuality to avoid victimization (Ellis, 2009), chronic vigilance and identity concealment as coping strategies may become stressful in themselves (Hatzenbuehler & Pachankis, 2016; Miller & Major, 2000), leading to negative judgements about the self, self-devaluation, and internal conflict (Meyer & Dean, 1998). Interestingly, internalized homophobia has been linked to a greater risk of unwanted sexual experiences (Salim et al., 2020) theorized to be due to internalized homophobia undermining the ability of sexual minority students to respond assertively to sexual threats and/or of potential perpetrators identifying this (Murchison et al., 2017).

According to MST, there are some group-level resources available to LGB individuals that may protect against minority stress (Meyer, 2003). For example, minority groups can provide a space free from victimization and offer social support if it occurs. In addition, sexual minority women report using lesbian and gay bars as safe spaces to avoid the unwanted advances of heterosexual men (Hequembourg & Brallier, 2009)—the effectiveness of which is seemingly confirmed by Murchison et al. (2017) who found that connection to the LGBTQ community was associated with a lower risk of unwanted sexual experiences. Given the alleged “climate of fear” (Ellis, 2009, p. 19), it is pertinent to understand how these resources are utilized by sexual minority students in protecting them from, and helping them to cope with, sexual harassment.

Thus, this study sought to further explore, qualitatively, the characteristics and consequences of everyday sexual harassment for female lesbian, bisexual, and queer (LBQ) students in the UK. Semistructured interviews were chosen as the most appropriate methodology for eliciting in-depth responses. Importantly too, compared to anonymous and/or quantitative/mixed methods, this approach ensured that the researchers could monitor the well-being of participants and that the participants felt heard and valued in giving their contribution (Beck, 2005; Hutchinson et al., 1994). The focus was on every day, low-level forms of harassment—too often, a neglected part of the continuum of sexual violence towards women (Kavanaugh, 2013) was excluded due to being “nearly universal among students who take part in sexualized social spaces” (Murchison et al., 2017, p. 225). However, given the known harmful consequences of everyday sexual harassment, and the growing need to challenge the normalization of everyday harassment (Hindes & Fileborn, 2022), examining these experiences is imperative. Thus this study sought to explore the under-studied topic of everyday sexual harassment in a sample of UK LGB university students at a time when sexual harassment on campuses is rife. This study sought to answer the questions: How do LGB young women experience everyday harassment, and what is the impact of this on them?

## Methods

### Participants and Recruitment

Participants were university students at a large campus University in the UK, recruited via snowballing after advertising on social media (Instagram). Those interested in participating were invited to contact the researchers. The inclusion criteria stipulated the following: (a) cisgender women, (b) identifying as lesbian, bisexual, or queer, (c) undergraduate students and (d) 18–24 years of age. Inclusion of trans women, trans feminine individuals, and nonbinary-identifying birth-assigned females was deemed beyond the remit of this particular study due to the anticipated breadth of experiences that may be identified. Additionally, any cisgender participants were excluded if they were currently undergoing treatment for trauma or other psychological effects related to experiencing sexual harassment, violence, abuse, or any other well-being issues. No incentive was offered for taking part.

Fifteen participants answered the initial recruitment advertisement and were sent the study information. Of these, thirteen participants expressed a continued interest. Interviews were conducted with nine cisgender women between the ages of 19 and 24 (*M* = 21, *SD* = 1.33) (see Table 1). Interviews were halted when it was felt that no new themes were being generated (Saunders et al., 2018). All participants identified as White, apart from one who identified as belonging to multiple ethnic groups.

**Table 1.** Participant Sexual Orientation and Relationship Status.

Pseudonym	Age	Sexual Orientation	Relationship Status
Danni	24	Bisexual	In a heterosexual relationship
Ellen	22	Bisexual	In a heterosexual relationship
Alex	21	Lesbian	In a same-sex relationship
Flo	21	Lesbian	In a same-sex relationship
Hattie	20	Lesbian	In a same-sex relationship
Indie	21	Lesbian	In a same-sex relationship
Bridget	20	Queer	In a same-sex relationship
Charlie	21	Queer	In a same-sex relationship
Grace	19	Queer	Single

### Procedure

After gaining the consent, participants were emailed with a list of definitions and examples of sexual harassment for reference and a link to an online survey in order to obtain demographic information. Once completed, the interview was scheduled. Interviews were conducted via Microsoft Teams, due to the COVID-19 pandemic precluding face-to-face interviews. Both the researchers had formal education in qualitative methods, and both were present during the interviews, with the consent of the participant. CC led the interview and GW observed (with camera off and muted) to ensure safeguarding. The interviews were conducted on a semistructured basis, each lasting between 25 and 40 min. Participants were assigned a pseudonym which their data would be stored under to ensure anonymity. Ethical approval was granted by Loughborough University (Human Participants) Sub-Committee (ref. 2466).

### Philosophical Framework

The research took an existential phenomenological approach (EPA) in order to describe participants’ core experiences (Churchill, 2022) and assumed a critical realist (CR) standpoint, a philosophical framework steadily gaining popularity within social scientific research (Fletcher, 2017). This approach is underpinned by the assumption that there is some authentic reality which acts as the foundation for knowledge (Stainton Rogers & Stainton Rogers, 1997). In line with this, a CR approach can allow marginalized groups to have their voices heard and validated (Lennox & Jurdi-Hage, 2017). Thus, participant experiences and understandings can be used to inform concrete policy recommendations addressing social issues (Fletcher, 2017).

### Researcher Position

In research involving sexual minority groups, it is essential to acknowledge the position of the researchers in relation to the participants (Bettinger, 2008). In the current study, the interviewing researcher (CC) provided an insider, or emic, perspective in terms peer, gender, and sexual minority status. This helped to establish rapport and facilitate trust building between them and the participants (Bettinger, 2010). To ensure rigour, both the lead researcher (GW) and the interviewing researcher were aware of the potential for bias due to the latter's insider status and the influence of their own experience on data analysis (Chavez, 2008). Thus, the interviewing researcher endeavoured to maintain enough distance to provide a balanced emic/insider and etic/outsider perspective, and debriefing/bracketing conversations were discussed between researchers after each interview. Given the diversity within sexual minority communities, this also helped to avoid assumptions of shared experience and allowed for an appreciation of the potential nuances in the experiences of participants with intersecting identities (Hayfield & Huxley, 2015).

### Measures

Consistent with the CR approach, the semistructured interview questions were developed (see Supplementary Material) to allow for flexibility in exploring the current literature and the potential for new ideas to emerge (Fletcher, 2017). The questions were designed to explore the characteristics and consequences of everyday sexual harassment perpetrated towards LBQ students. Participants were asked to answer questions with examples of gender harassment (by far the most common, everyday form of harassment; Bondestam & Lundqvist, 2020; Gelfand et al., 1995) and unwanted sexual attention (as behaviors in this category are most recognizable to students as harassing; del Carmen Herrera et al., 2017). Due to ethical concerns related to the nature of the topic and the limited experience of the interviewing researcher in this specific area, sexual coercion was excluded from the research as it is generally considered a more severe form of harassment. Given that students do not always label unwanted, unpleasant acts as sexual harassment (Vohlídalová, 2011) definitions were provided alongside examples of gender harassment and unwanted sexual attention. Respectively, these were taken from Gelfand et al.'s (1995) model and from the NUS survey (Smith, 2010) which surveyed the prevalence of the UK students’ experiences of sexual harassment and violence in further education (see Supplementary Material). The interview questions also aimed to explore minority stress processes in relation to sexual harassment.

### Data Analysis

The interview data were analysed by following Braun and Clarke's (2006) steps of thematic analysis. Consistent with CR, the analysis was predominantly researcher driven. Throughout the process, the researchers played an active role, searching for meaning and patterns across the entire data set. (1) The first phase of analysis involved familiarizing onself with the data and transcribing the interviews verbatim. The interviewing researcher transcribed the interviews, and both researchers familiarized themselves with the transcripts. (2) Secondly, with the CR framework in mind, the subsequent coding process was deductive (drawing on existing sexual harassment theory and MST) while also being flexible to allow for new or unexpected patterns to be identified (Fletcher, 2017). The data were manually coded after all of the interviews had been conducted and were coded at the semantic level where participant accounts were taken at face value and were the focus of the analysis (Braun & Clarke, 2006). The interviewing researcher coded the data first, followed by an independent review by the lead researcher. (3) Next, the codes were sorted into potential themes by the interviewing researcher before (4) returning to the transcripts with the lead researcher to consider whether these ‘worked’ in relation to the data (Braun & Clarke, 2006). (5) The fifth stage involved defining and refining themes based on the further review performed by the lead researcher, ensuring they did not overlap and that individual themes were not too diverse or complex. Agreement between the researchers was very close (94%), and minor deliberations were related to the subthemes only and were resolved through discussion. A final revision of the structure of themes was undertaken by the interviewing researcher (6) to produce the results that follow.

## Findings and Discussion

Three overarching themes were constructed: (1) the paradox of men's unwanted sexual attention, (2) the negative impact on relationships: the intra- and interpersonal, and (3) the LGBTQ* community as a refuge.

### Theme 1: The Paradox of Men's Unwanted Sexual Attention

Many of the women reported receiving unwanted sexual attention exclusively perpetrated by heterosexual men. There were two paradoxical ways in which this form of sexual harassment manifested. The women were (a) marginalized by men and (b) desired by men.

#### (a) ‘Lesbian sex is not real sex’: Marginalized by men

Based on their gender and sexuality, the women described being put-down and made to feel inferior to heterosexual women and men. This double discrimination was evident in Ellen's reflections on the harassment of LBQ women:

[…] obviously being a gay woman is another added kind of thing, and they [men] may be kind of more likely to subject them to harassment than just being a woman, a straight woman, I think it's probably a similar thing, I think it's all about superiority (Ellen)

Feeling inferior was also alluded to when Hattie recalled a group of men asking invasive questions about her sex life: “because obviously it's a marginalized group anyway, and you’re a woman, and then, it's like they’re tryna [sic] make you feel smaller than you are” (Hattie). The double discrimination based on the gender and sexuality mirrors that reported by lesbian and bisexual women in Hequembourg and Brailler's (2009) focus group study.

Many of the women in the current study reported unwanted sexual attention similar to the well-documented experiences of heterosexual female students such as catcalling, groping, and general sexual comments (Smith, 2010). Since these experiences often occurred before sexuality disclosure, the women may have been perceived as heterosexual, or perhaps simply being a woman rendered them a suitable target for harassment. According to gender-based explanations of sexual harassment (Berdahl, 2007; MacKinnon, 1979), these behaviors act to marginalize all women from society and thus the women's sexuality may not have been particularly relevant to perpetrators.

However, the harasser's perception of the women's sexuality did seem important for the type of harassment perpetrated. Upon disclosure of LBQ status, several participants described men's “intimate,” “personal,” and “inappropriate” questions about the women's romantic relationships. In a recent study, such inappropriate curiosity has been referred to as the “novelty treatment” (Serpe et al., 2020, p. 466) where heterosexual men base their questions on stereotypes, such as sexual minority women being overly sexual people or being greedy. For some of the women in the current study, these questions also led to erasure and their sexual experiences being judged as “not really having sex” (Bridget). This dismissal was described in Alex's account of an incident which occurred whilst she was working on a campus:

[…] they then made jokes, continued to say lesbian sex was not real sex, on a number of times I continued to say to this person ‘Please can you stop making these jokes?’, and then the other guys we were all with continued to join in with the jokes and say how lesbians have clearly not met the right man, and that basically we didn’t know what we were doing (Alex)

Alex explained that these experiences can lead to the silencing of LBQ women: “it's a shame, that if you’re a lesbian or a bisexual person, that you can’t talk about sex the same way that a straight person would be able to do” (Alex).

Some participants felt that the sexual harassment of LBQ women could take the form of homophobia. This understanding of sexual harassment was reflected in Charlie's account of an incident in a nightclub:

[…] someone came up the stairs to the top area, erm, and looked, and I sort of had my arm kind of round her and we were sat in the booth and he just looked, and he went “faggot” right at me (Charlie)

It is noted that this event does not exactly fit the definitions of sexual harassment utilized by the current study. However, as Fileborn (2014) highlights, for sexual minority individuals, unwanted sexual attention and homophobic violence often overlap and intersect. What is important is that this experience was salient for Charlie and exposes the homophobic attitudes and behaviors which continue to marginalize LBQ students in public spaces.

#### (b) ‘I got the lesbian in bed!’: Desired by men

Paradoxically, whilst the women were marginalized because of their sexual identity, they seemed to be especially desired by heterosexual men for this reason. Based on stereotypes of LBQ women being inherently sexual people, men eroticised female same-sexual behaviors such as kissing and holding hands. Desire and fetishization were described negatively and regarded as “objectifying,” reflected in the quotes: “people would like shout like derogatory stuff at us […] being like ‘oh show us a kiss!’” (Ellen) and “they see us as a form of their own entertainment and their own joy rather than two people” (Bridget). Danni described the unwanted male attention she received after kissing another woman in a nightclub: “all these boys like surrounded us, taking videos of me, taking pictures, it was so disgusting, and they were all like yelling stuff” (Danni). These accounts point to the sexual performances that LBQ women are often requested to engage in for the gratification of male audiences (Boyer & Galupo, 2015). Boyer and Galupo (2015) propose that same-sex eroticism between women may either be considered a stunt or that heterosexual men may expect LBQ women to engage in performances to authenticate their sexuality. This behavior assumes that heterosexuality is the only authentic sexual identity and thus acts to further marginalize LBQ women. These observations confirm the continued existence of heterosexist attitudes on the university campus and in public places.

Participants described men's unwanted sexual attention taking the form of propositions, reflected in the quotes: “[men] being like ‘I could turn you’ like ‘you haven’t slept with a boy why don’t you try it?’” (Flo) and “people have tried to make moves on [my girlfriend] because she's a lesbian and she has not slept with any boys ever” (Alex). Bridget described a sexual encounter with a male student in her university accommodation and explained her understanding of men's sexual pursuit of LBQ women:

[…] when they know that you’re not straight, it becomes more of a game, and that's what I’ve experienced, it worsens because suddenly they’re like ‘If I can get her in bed, I got the lesbian in bed’ […] I slept with someone and the first thing he said after was ‘I got the lesbian in bed!’ […] until coming to Uni, unfortunately, I’d never viewed being er a queer woman as, like, a challenge (Bridget)

The notion of challenge and achievement was further revealed in several participants’ accounts of men's requests for threesomes. Male involvement in female same-sex relationships was thought to be fetishized, and same-sex partners used as sexual objects to enhance men's social status amongst other men: “obviously having a threesome with two bisexuals is seen as something to one-up his mates and stuff like that, its seen as quite a cool thing to have” (Ellen). Much of this harassment was explained by men “showing off” to their friends, which may reflect the competitive and sexual nature of ‘lad culture’ which has pervaded UK university campuses within the last decade (Craig, 2016; Phipps & Young, 2013). Female students define ‘lad culture’ as a pack mentality involving men's objectification of women and use of “banter” to disguise and trivialize misogyny, sexism, and homophobia (Phipps & Young, 2013). In the social sphere of university life, this group mentality was seen to perpetuate gender harassment and unwanted sexual attention, most commonly in the forms of misogynistic jokes and physical molestation. Indeed, some of the women in the current study explicitly referred to ‘lad culture’ or “laddish” behavior (Alex, Flo) as an explanation of the unwanted sexual attention they received from groups of male students. The existence of ‘lad culture’ is problematic for campus culture as a whole since the two are often considered to be strongly connected, even inseparable (Phipps & Young, 2013). In student communities where ‘lad culture’ is dominant, sexual boundaries are likely to be routinely crossed creating an environment conducive to sexual assault and rape (Stenson, 2020) where women have to engage in ‘safety behavior work’ to keep themselves safe (Bovill et al., 2022). Thus its enduring presence is worrying and warrants attention from the university.

Whilst being marginalized and desired by men are paradoxical experiences, participants saw both types of unwanted sexual attention as equally unpleasant and degrading.

### Theme 2: The Negative Impact on Relationships: Intra- and Interpersonal

Experiencing sexual harassment had negative impacts for how the women related both to (a) themselves and (b) to other people.

#### (a) ‘I’ve had to adapt who I am’: Relating to the self

The sexual harassment endured by the women affected how they related to themselves, their sexual identity, and their emotional responses to that harassment. Many of the participants were made to feel “embarrassed” and “uncomfortable” by sexual propositions and invasive questions about their sex lives. There was often a sense of confusion in how the women related to these emotions. Flo described how she felt after being asked to participate in a threesome: “I just was a bit taken aback, I didn’t expect it at all, and I was almost a bit embarrassed, I don’t know why I was embarrassed” (Flo). Similarly, Alex's response to homophobic comments and being asked inappropriate questions was one of internal discomfort and doubt: “on a number of occasions I asked him to stop because it made me feel uncomfortable and made me question how I was feeling towards things” (Alex).

The internalization and self-doubt experienced by the women extended beyond their immediate reactions. Speaking in general about her experiences of sexual harassment, Bridget described her struggle in relation to her sexual identity:

It's made me question a lot of things, like if I wasn’t gay would I be targeted this much […] if I wasn’t holding hands with my girlfriend would they have done that too, is it just because I’m gay […] it impacts me on my thoughts, I had an awful like first few months at Uni, I was really hating on the fact that I was gay, I was hating myself for it, and I shouldn’t have (Bridget)

This internalized homophobia was more explicitly referred to by Grace: “I think it can kind of bring about internalized homophobia […] feeling more shame about your sexuality, and feeling, othered, I guess compared to people who are straight” (Grace). These accounts demonstrate the interrelatedness of minority stress processes in that internalized homophobia and perceptions of LGB stigma can result from external prejudice events (Meyer, 2003), in this case, from the sexual harassment based on the women's LBQ status.

Generally, there is a lack of qualitative inquiry into sexual minority women's experiences of internalized homophobia. However one study did report that experiencing internalized homophobia meant that women in same-sex couples struggled to accept their sexual identity (Rostosky et al., 2007), and a more recent study has suggested that this may impact upon self-esteem (Bridge et al., 2022a). Similarly, the current study found that the internalization and expectation of prejudice experiences could manifest as an unstable sense of self:

I’ve had to adapt who I am, and I’ve tried to go back to it, I’ve tried to not care, but again with the whole physical thing, if it became physical, I wouldn’t be able to defend me or my girlfriend, so it's hard (Bridget)

Quantitative studies have revealed that internalized homophobia can predict psychological distress in lesbian and bisexual women (Kaysen et al., 2014; Szymanski & Kashubeck-West, 2008). Whilst many of the women mentioned the psychological impacts of sexual harassment, the mental health problems postulated by MST to result from prejudice events (Meyer, 2003) were not explicitly discussed. This may have been because the research excluded women currently undergoing treatment for any psychological effects relating to experiencing sexual harassment. Alternatively, for some women, everyday sexual harassment experiences may have become so normalized that they do not cause major psychological distress (Mellgren et al., 2018). These possibilities warrant further qualitative study.

#### (b) ‘We don’t hold hands anymore’: Relating to other people

The sexual harassment experienced by the women had negative impacts on several interpersonal relationships. For some, the sexual harassment perpetrated by acquaintances acted as a barrier to forming and maintaining friendships at university. Flo described being sexualized by her flatmates in her accommodation, and the impact this had for her relationship with them:

I did really like my flatmates, and then they started saying stuff like that [‘have you been with a guy?’, ‘do you wanna sleep with me?’] and I was like oh, that's weird […] I think like maybe if I hadn’t experienced that, I’d be more willing to have a house with like my flatmates (Flo)

Participants reported becoming more wary of others and hypervigilant of their intentions in conversations around sex and relationships. This was revealed by Bridget: “I always get more defensive with men, because obviously now I have that point of view that I’m being objectified” (Bridget). Many of the women felt as though they may have to conceal their sexual identity to avoid being fetishized and asked invasive questions: “it kind of makes you not wanna [sic] tell people that you are part of that community, because you don’t want people to, yeah oversexualize you” (Danni). McLean's (2007) interview data echo these sentiments; identity disclosure for sexual minority women involved a lengthy and stressful deliberation process, weighing up the benefits and potential costs, including being objectified and sexualized, as mentioned by the women in the current study.

The sexual harassment faced by LBQ women also had implications for their romantic relationships. Ellen spoke about the destructive impact that experiencing sexual harassment had on a former relationship:

[…] me and my girlfriend […] well my ex-girlfriend, we aren’t together anymore, cus [sic] she basically, further down the line she ended up cheating on me, and the reason she gave was because, with a guy she didn’t see the same abuse as she did with me (Ellen)

Ellen, who is bisexual, felt that the harassment she faced whilst in a same-sex relationship would deter her from having another female partner in the future.

Many of the women spoke of concealing their sexual identity by avoiding kissing or holding hands with their female partner. Due to actual and feared victimization experiences, some described how the reluctance to display same-sex affection led to restrictions in how they used public space, both on and off campus. This is exemplified by the following quote:

You’re sort of made to feel like you can’t then, be visible, like visibly gay in public […] I always am really wary about holding hands or anything, because it's sort of like, you see a lot in the news, especially like that couple who got beaten up on a bus […] obviously I don’t wanna be in that situation, so I will just avoid it (Hattie)

Similarly, in Rostosky et al.'s (2007) qualitative investigation of minority stress, same- sex couples described carefully monitoring their behaviors to ensure they were not seen as a couple to avoid verbal attacks and unwanted stares. The women's concealment of identity and avoidance of situations constitute suppressive coping strategies (Heppner et al., 1995). This form of coping prevents the resolution of stressful situations and is potentially maladaptive. It has been found to predict psychological distress in sexual minority women experiencing prejudice events and internalized homophobia (Szymanski & Henrichs-Beck, 2014). These findings suggest that it may be pertinent for future research to examine the coping strategies used by sexual minority women. University policy makers should be aware of, and facilitate, the coping strategies which prevail as most adaptive in order to help to ameliorate the psychological and interpersonal distress discussed by some of the women. However, it is recognized that the primary prevention of sexual harassment should be the main priority, and it should not be the responsibility of LBQ women to better cope with harassment.

### Theme 3: The LGBTQ* Community as a Refuge

The LGBTQ* community was seen as a refuge which was (a) a harassment-free community and (b) an affirming community.

#### (a) ‘In a little bubble’: A harassment-Free community

For many of the women exclusive and inclusive, LGBTQ* communities were regarded as largely free from sexual harassment. The women talked about physical safe spaces where they felt more comfortable being open about their sexual identity without the risk of being sexualized or harassed:

[…] we’ve been to obviously lesbian bars together because there, you’re not going to experience that, and no-one's going to look at you and be like ‘er’ or ‘omg that's so hot’, no one really makes it weird (Alex)

This mirrors the accounts of sexual minority women in previous research who used lesbian bars to avoid undesirable interactions with heterosexual men (Hequembourg & Brallier, 2009). Conversely, Fileborn (2014) reported that unwanted sexual attention occurred between sexual minority individuals within LGBTQ specific venues. Despite this, Fileborn's participants still viewed gay and lesbian bars as safe spaces; ‘safety' was the ability to express sexual identity rather than the total absence of sexual harassment conveyed by women in the current study.

Some of the women belonged to the university football club. This was a space in which its members felt “accepted,” “welcomed,” and “comfortable.” Traditionally masculine sports including football have repeatedly been identified as safe spaces for sexual minority women (NUS, 2014; Pay, 2017; Phipps, 2020). Despite findings to suggest that the male gaze may be replaced by the lesbian gaze within these arenas (Pay, 2017), this was not the case for the women in the current study. Indie believed that being part of the football “bubble” was one of the main reasons she had never been sexually harassed. For Flo, belonging to this social subgroup was also seen to protect its members against sexual harassment from outside the club: “[…] it's almost like you don’t have to go and socialize with other people, if you weren’t in that group, you may be more […] vulnerable to it”. Interestingly, a recent study found that women who were part of university football and rugby teams often experienced sexual harassment from outside of these communities (Phipps, 2020). Harassment was based on presumed nonheterosexuality due to stereotypes about traditionally masculine sport involvement and took the form of invasive questions about sexuality and sex lives. Thus Phipps (2020) suggested that university sport may only be inclusive within teams. On the contrary, footballers in the current study regarded their club as wholly protective of sexual harassment. However in relation to attending general LGBTQ* groups and events run by the student's union, Hattie was concerned that these might make her more vulnerable to sexual harassment from outside the community:

[…] I used to go to the LGBT society ‘Out’ night, and a lot of the time, in my mind, I’d be thinking, would people then see me, as a woman coming out of this gay little bar going on, and then, that would be another reason to harass me as I’m walking home (Hattie)

Charlie provided an account of unwanted sexual attention from within the LGBTQ* community. She described a bisexual woman making a sexual advance towards her and her girlfriend. Notably this encounter was not labelled as sexual harassment since the woman was not persistent. Other participants also felt that a female perpetrator would be less persistent and therefore less threatening than a heterosexual male perpetrator: “the target would probably feel safer to erm, not, resist isn’t the word but, more likely to assert themselves” (Grace). Similar sentiments were evident as participants spoke about being less easily overpowered by women and feeling more able to defend themselves.

Bridget made an interesting observation in explaining the absence of female perpetration: “if they [women] were suddenly doing it, and the person they were trying to talk to says no, erm its more of a reaction of ‘Oh no, I’m causing her to feel what I have felt, I need to stop’”. This empathy attributed to other women was a common explanation the participants gave for women not harassing each other; heterosexual or LBQ. Indeed, research has shown that the gender differences in harassment perpetration can be explained by differences in empathy (Diehl et al., 2014). This may support the use of sexual harassment prevention interventions utilizing perspective taking which aim to increase potential perpetrators’ empathy for the negative consequences of sexual harassment (Moore & Mennicke, 2020).

#### (b) ‘I will not take it from you’: An affirming community

Some women spoke of the value of having friends belonging to the LGBTQ* community in terms of comfort and support following sexual harassment. Friends were seen as a coping resource; groups who could empathize and validate negative emotions and experiences:

I have a group of friends that are all from the LGBTQ community and we all experience these same things that I do and its really really helpful to talk to them about it and share experiences and ask for advice or even, just talk about it, it's really helpful to be part of that community and be understood by similar people (Ellen)

In this way, discussing sexual harassment created a sense of shared experience and provided a platform for the women to bond with other members of the LGBTQ* community. According to MST, minority group affiliations act as a coping resource as members can help to reappraise stressful situations making them less damaging to mental health (Meyer, 2003). Empirical research also shows that social support from within LGBTQ* communities can protect against the psychological distress caused by internalized homophobia (Szymanski & Kashubeck-West, 2008) and can support self-esteem (Bridge et al., 2022b). Considering the internalized homophobia and psychological impact described by the women, it is important for the university to create opportunities for LBQ students to build alliances within the community. One example is the university's LGBT + association which some of the women were actively involved with. The affirmation provided by the community helped the women to feel confident in their sexuality which instilled in them a sense of resilience in their response to sexual harassment. They felt able to respond assertively and empowered to challenge behaviors on an individual level:

[…] coming here and meeting my current girlfriend and being taught what an awesome community it is, it's meant that I haven’t changed negatively, I’ve changed positively wanting to educate people about it […] I wanted to tell people, which is kind of why I’ve done this, to tell people that like we do get harassed and it's not okay and that, it should change because it's not talked about enough (Bridget)

Calling-out sexual harassment and discrimination—when safe to do so—was also an important coping strategy for bisexual women in a recent study (Serpe et al., 2020). It embodies a reflective coping style (Heppner et al., 1995) intended to produce changes in the stressful situation. In this case, the stressful situation is a heterosexist society which enables the sexual harassment of LBQ women to persist. In the current study, only a minority of the women chose to confront harassers. However, this form of coping is thought to result in fewer negative mental health outcomes compared to passive and suppressive strategies such as avoidance and concealment of identity (Heppner et al., 1995; Lehavot, 2012). This potentially demonstrates the value of university LGBTQ*-affirming communities which afford LBQ women the confidence to call-out harassment.

### Limitations and Future Direction

The current study provides valuable insight into the lived sexual harassment experiences of LBQ women. However, there are five notable limitations. Firstly, all but one of the participants were White meaning that the experiences of sexual harassment captured in the data may not represent those of LBQ women of color. Given that women of color often receive sexual harassment based on racial stereotypes (Richardson & Taylor, 2009), future research should endeavour to explore the manifestations of sexual harassment in which gender and sexuality intersect with race. Secondly, it was beyond the scope of the current study to provide a detailed exploration of each woman's individual sexual identity and how this then related to sexual harassment experiences. However it is recognized that sexual minority women should not be considered a homogenous group and that their sexuality may affect their experiences. Indeed, it has been suggested that some women with bisexual identities feel less connected to LGBTQ* communities than lesbian women (Balsam & Mohr, 2007). Therefore future qualitative research may provide insights into how sexual harassment is experienced and coped with depending on women's specific sexuality. Relatedly, this study only included cisgender women. Future work will benefit from exploring the everyday sexual harassment experiences of trans* women in order to explore how harassment based on patriarchal notions of power is experienced by all female-identifying people. Fourthly, these interviews were conducted with young women attending University and as such will not represent the changing manifestations and experiences of harassment across the life-course. Older LGB women's experiences therefore also need to be explored. Finally, from a methodological standpoint, to reduce any possibility of researcher bias, it would have been beneficial to check themes and interpretation with members of the University LBQ community. Future work should seek to ensure that beyond insider researcher status and co-researcher checking, member checking forms an integral part of interpretation.

### Implications

In line with the critical realist framework, the findings of the current study have the potential to lead to definitive action and solutions to address sexual harassment (Fletcher, 2017). The narratives of the women provide clear evidence of minority stress, and these are explicitly linked to their sexuality and experiences of harassment (internalized homophobia, intimate behavior modification, concealment of identity, and feeling victimized by unwanted, heterosexist offensive attention). It is hoped that the findings of this study could be used to support the inclusion of sexual minority specific messages in university sexual violence awareness and prevention campaigns. The women's accounts illustrate certain forms of verbal unwanted sexual attention which are exclusively perpetrated towards LBQ women. Awareness and prevention campaigns aimed at general audiences should include descriptions of these forms of harassment as it is essential for programs to include accounts of the lived experiences of potential victims/survivors (Kavanaugh, 2013). The current study also indicates the role of empathy in stopping women from harassing each other. Thus prevention programs aimed at potential perpetrators should include perspective taking and illuminate the negative consequences of sexual harassment (Moore & Mennicke, 2020). Again, these interventions should also include sexuality-specific messages about the consequences of harassment for LBQ women such as internalized homophobia, fear of revealing sexual identity, and relationship difficulties.

It is evident that heterosexist and homophobic attitudes still exist on the university campus. These were often attributed to ‘lad culture’ and were seen to motivate the sexual harassment of LBQ women. Therefore, despite the reported improvement towards the elimination of problematic ‘lad culture’, there is still progress to be made (Stenson, 2020). Stenson proposes a multilevel transformation effort where student activists use their experiences of ‘lad culture’ to help develop university guidelines and the institution is then responsible for disseminating and reinforcing the guidelines to the larger student population. The accounts gained in the current study may be used for such purposes.

The priorities of the aforementioned implications are the primary prevention of sexual harassment and visible challenging of heterosexism. This may be achieved by increasing bystander training (Davies et al., 2022) but also potential perpetrators’ awareness of harassing behaviors and the consequences for the victims. Ideally, this should be undertaken in conjunction with mandatory education for all students on heteronormativity and feminism—since the content, and context, of UK Higher Education remains dominated by White males (Morris et al., 2022). Since the findings also suggest that LBQ women are still being sexually harassed, the university should provide safe spaces and promote accepting societies and sport clubs. This will enable LBQ women to build social support networks which may help them to feel affirmed and potentially foster more adaptive coping styles.

## Conclusion

The aim of the current study was to add to the sexual harassment literature by using qualitative methods to explore the characteristics and consequences of sexual harassment for female LBQ students. The women in the study reported enduring sexual harassment representing discrimination based on both their gender and sexual minority status, including invasive questions about their sex lives, relentless requests for threesomes, and having their relationships eroticized. These experiences support the inclusion of sexuality-specific messages in sexual harassment prevention campaigns run by the university and in challenging the continuing ‘lad culture’ on UK campuses. Minority stress was evident, with women expecting harassment, concealing their sexual identity to avoid such harassment, internalizing these experiences, and enduring various levels of psychological distress. Importantly, women found solace in inclusive and exclusive LGBTQ* communities which provided safe-spaces and afforded some women the confidence to foster adaptive strategies to cope with sexual harassment. While this is very positive, it should be noted that not all LBQ women will choose to identify with such spaces/communities and so many women may not have easy access to support. This adds further weight to the need for wider, generalized education to help combat the harassment experienced by LBQ women and indeed all women. Future studies should address the limitations of the current research by exploring the intersections of gender identity, sexuality, and race in women's experiences of sexual harassment and how experiences differ depending on women's specific sexual orientations. In addition, support interventions should not be framed around LBQ spaces/identities exclusively since this may serve to exclude those who not identify with such.

## Supplemental Material

sj-docx-1-vaw-10.1177_10778012231166399 - Supplemental material for ‘Show Us a Kiss!’: The Everyday Sexual Harassment Experiences of Female Lesbian, Bisexual, and Queer Students Supplemental material, sj-docx-1-vaw-10.1177_10778012231166399 for ‘Show Us a Kiss!’: The Everyday Sexual Harassment Experiences of Female Lesbian, Bisexual, and Queer Students by Gemma L. Witcomb and Charlotte Cooper in Violence Against Women
