# Supplementary material for: ‘Show Us a Kiss!’: The Everyday Sexual Harassment Experiences of Female Lesbian, Bisexual, and Queer Students
Source: Violence Against Women. 2023 Apr 5;30(11):3055–76. doi: 10.1177/10778012231166399 (PMC11316343; doi:10.1177/10778012231166399)
Supplement: sj-docx-1-vaw-10.1177_10778012231166399 - Supplemental material for ‘Show Us a Kiss!’: The Everyday Sexual Harassment Experiences of Female Lesbian, Bisexual, and Queer Students [file sj-docx-1-vaw-10.1177_10778012231166399.docx]

**SUPPLEMENTARY MATERIAL**

**Information given to participants**

These questions will focus on your experiences of ‘everyday’ low-level sexual harassment. This is defined as **unwanted sexual attention** and can include verbal and non-verbal behaviours which can create a hostile environment.

Examples include:

1. **Verbal harassment**

- Someone making comments with a sexual overtone that made you feel uncomfortable
- Someone wolf whistling, catcalling or making noises with sexual overtones
- Someone asking questions about your sex or romantic life when it was clearly irrelevant and none of their business
- Someone asking you questions about your sexuality when it was clearly irrelevant or none of their business

1. **Physical/non-verbal harassment**

- Someone exposing their sexual organs to you when you did not agree to see them
- Someone groping, pinching or smacking your bottom when you did not agree to them doing so
- Someone groping, pinching or touching your breasts when you did not agree to them doing so
- Someone lifting up your skirt in public without you agreeing

1. **Gender Harassment** - behaviours that communicate derogatory attitude about women such as sexist jokes, gestures, or images – examples from Gelfand et al., 1995.

- Someone telling you suggestive stories
- Someone making crude sexual remarks
- Someone showing you sexist material
- Being treated differently because of your gender
- Making sexist remarks

In this study we are not referring to severe forms of harassment or assault such as

**sexual coercion** (explicit or subtle threats or bribes related to sexual co-operation and negative consequences for not co-operating e.g. been in line for a promotion, then your boss asks you on a date to which you decline, and you lose your promotion) and **sexual assault** (e.g. rape, attempted rape, sexual violence).

All questions will refer to the period of time since you started university and can relate to any aspect of life i.e. inside or outside the university setting

**Semi-structured interview questions**

1. Since starting university, have you been subjected to any forms of sexual harassment like the examples I have given? *If no, move to question 4.*
2. Can you estimate how many times you have been subjected to any form of sexual harassment since starting university?
3. Please can you describe in detail one example of when you have experienced sexual harassment
4. What were the characteristics of the harassment i.e. what was done or said?
5. Where did it occur?
6. Who was the perpetrator?
7. What do you think the motivations behind the harassment were?
8. How did it make you feel?
9. How did you react at the time?
10. How do you feel about it now?
11. What are the short- and long-term effects of the experience? *(Social / Psychological / Emotional / Behavioural / Academic)*
12. In more general terms, in what types of places have you experienced sexual harassment, either directed at yourself, or at another woman who is lesbian, bisexual or queer?
13. In your experience, what are the most common types of sexual harassment that LBQ females experience?
14. Do you feel that being female influences the amount or types of sexual harassment you, or other women, are subjected to? *If yes, how?*
15. Do you feel that being part of the LGBTQ* community influences the amount or types or sexual harassment you, or other LBQ women, are subjected to? *If yes, how?*
16. What do you think the main motivations behind sexual harassment directed to LBQ women are? *Do these depend on the context? How?*
17. What do you think are the effects of your, or other LBQ females’ experiences of unwanted sexual attention?
18. Social
19. Psychological
20. Emotional
21. Behavioural
22. Academic
23. Are these effects influenced by gender or sexual orientation?
